# Performance Monitoring Applied to System Supervision

**DOI:** 10.3389/fnhum.2017.00360

**Published:** 2017-07-11

**Authors:** Bertille Somon, Aurélie Campagne, Arnaud Delorme, Bruno Berberian

**Affiliations:** ^1^ONERA, Information Processing and Systems Department Salon Air, France; ^2^Univ. Grenoble Alpes, CNRS, LPNC UMR 5105 Grenoble, France; ^3^Centre de Recherche Cerveau & Cognition, Pavillon Baudot, Hopital Purpan, BP-25202 Toulouse, France; ^4^Swartz Center for Computational Neurosciences, University of California, San Diego San Diego, La Jolla, CA, United States

**Keywords:** Performance monitoring, error-related negativity, feedback-related negativity, neuroergonomics, error detection, system monitoring, Out-of-the-loop, mind-wandering

## Abstract

Nowadays, automation is present in every aspect of our daily life and has some benefits. Nonetheless, empirical data suggest that traditional automation has many negative performance and safety consequences as it changed task performers into task supervisors. In this context, we propose to use recent insights into the anatomical and neurophysiological substrates of action monitoring in humans, to help further characterize performance monitoring during system supervision. Error monitoring is critical for humans to learn from the consequences of their actions. A wide variety of studies have shown that the error monitoring system is involved not only in our own errors, but also in the errors of others. We hypothesize that the neurobiological correlates of the self-performance monitoring activity can be applied to system supervision. At a larger scale, a better understanding of system supervision may allow its negative effects to be anticipated or even countered. This review is divided into three main parts. First, we assess the neurophysiological correlates of self-performance monitoring and their characteristics during error execution. Then, we extend these results to include performance monitoring and error observation of others or of systems. Finally, we provide further directions in the study of system supervision and assess the limits preventing us from studying a well-known phenomenon: the Out-Of-the-Loop (OOL) performance problem.

## 1. Automation and supervision difficulty

In the past decades, automation has become increasingly present in our daily life. Whether at work or at home, we interact more and more with sophisticated automated computer systems and software designed to assist us in our daily activities. More radical changes are anticipated in the future, as computers grow in power, speed and “intelligence”. We have usually focused on the perceived benefits of automation. Automation has enabled processes to become faster (e.g., traveling by plane), easier (e.g., in industrial production lines) and safer (e.g., automated processes in cars) to use (Kaber et al., 2000). However, these advances mask the fact that automation does not replace human activity, but rather modifies it, often in ways unintended and unanticipated by system designers (Carmody and Gluckman, 1993; Parasuraman et al., 2000). People who were previously performing manual activities have now moved on to more supervisory tasks (Sheridan, 1992, 1997), requiring high levels of awareness over long periods of time (Sheridan and Parasuraman, 2005). Bainbridge (1983) stated that There are two general categories of task left for an operator in an automated system […] to monitor […] or to take over.” This change in activity has led to new cognitive dysfunctions: a loss of operator situational awareness with respect to the automated system (Endsley, 1996), over-reliance toward highly reliable automated systems (the complacency phenomenon; Parasuraman et al., 1993), a decrease in vigilance (Moray and Inagaki, 2000; Sheridan and Parasuraman, 2005) and a decline in human performance (e.g., decision biases and failures of monitoring; Wiener, 1988; Parasuraman and Riley, 1997). This set of difficulties is called the Out-Of-the-Loop (OOL) phenomenon. Given the role of supervision in human-system interactions, and the fact that it triggers new dysfunctions, research about performance monitoring in an automated environment seems relevant.

The performance monitoring system is critical for successful goal directed behavior, given that human beings learn from the consequences of their actions (Holroyd and Coles, 2002). It was defined recently as “[…] a set of cognitive and affective functions determining whether adaptive control is needed and, if so, which type and magnitude is required.” (Ullsperger et al., 2014b). Thus, it has been widely studied using several experimental protocols–the Eriksen flanker attention task (Falkenstein et al., 2000; Scheffers and Coles, 2000; Roger et al., 2010), d2 selective attention test (Ora et al., 2015), Stroop task (Carter and van Veen, 2007), Go/NoGo paradigm (Vidal et al., 2000; Bates et al., 2005), Simon task (Bonini et al., 2014; Amiez et al., 2016), video watching (Desmet et al., 2014), and time estimation task (Miltner et al., 1997)–and using various measurement methods, including reaction time (Rabbitt, 1966a,b), electroencephalography (EEG; Falkenstein et al., 1991; Gehring et al., 1993; Ullsperger et al., 2014b), functional magnetic resonance imaging (fMRI; Botvinick et al., 1999; Carter and van Veen, 2007) and eye-tracking (Braem et al., 2015).

Self-performance monitoring is a process that is well understood in theory (Botvinick et al., 1999; Holroyd and Coles, 2002; Alexander and Brown, 2011) and is applied in varied contexts, such as aviation (Shappell et al., 2007), or medicine (Gehring et al., 2000; Taylor et al., 2007). In contrast, we have little knowledge about the processes involved in error monitoring of others' actions (van Schie et al., 2004) and in error monitoring of a system's actions (i.e., system supervision; Padrão et al., 2016). They are no less important to our interactions with the outside world, since they allow us to anticipate others behavior and react to it.

The purpose of this review is to summarize our knowledge on performance monitoring (see Taylor et al., 2007; Hoffmann and Falkenstein, 2012; Johnson and Gulbinaite, 2013, for other reviews) and, more specifically, as it relates to system supervision. Automation supervision becomes a daily routine for a lot of people, but after decades of research (from Bainbridge, 1983 to Baxter et al., 2012), the neural bases of this process have not been fully characterized and the cognitive deficits associated with system performance monitoring are still unclear.

## 2. Performance monitoring: a well-known mechanism

### 2.1. Performance monitoring system: first evidence with the error-related negativity (ERN) component

The existence of an internal process associated with monitoring our own errors was first suggested by Rabbitt (1966a). He showed that external cues had no effect on error-correction latencies in a 10-choice reaction task, and that subjects were still able to correct their own errors with a 100% rate without needing any feedback: even without error feedback, they knew that it was an erroneous trial.

The first EEG evidences associated with an internal error-monitoring system were reported by Gehring et al. (1990, 1993) and Falkenstein et al. (1991). In event-related potential (ERP) studies, these authors identified a component associated with participants' errors during choice reaction tasks: the *error negativity* (Ne; Falkenstein et al., 1991), or more commonly called the *error-related negativity* (ERN; Gehring et al., 1993). The ERN is a negative ERP time-locked to the participant's response errors, which starts around 6 ms before the response and peaks in the fronto-central region (maximum at FCz) around 80 ms after the erroneous response–or around 100 ms after electromyographic onset of response error (Dehaene et al., 1994; Vidal et al., 2000; Ullsperger et al., 2014a). The amplitude of the ERN varies positively with a speed-accuracy gradient: the more the participant is asked to be accurate (less speed) the more the amplitude of the ERN increases–and vice versa (Gehring et al., 1993). This is the speed-accuracy trade-off. The amplitude of the ERN is also correlated with correction speed, probability of correction, future errors and post-error slowing (Rodríguez-Fornells et al., 2002; Debener, 2005). Based on these results, the ERN is thought to reflect an automatic mismatch between the overt response and the outcome of the response selection process, and is an important neurophysiological marker of online performance monitoring (Falkenstein et al., 1991; Gehring et al., 1993). In addition, Gehring et al. (1993) suggested that the ERN represents error compensation and correction, and may play a role in stopping errors from happening while they are happening: it brakes the erroneous response. However, using electromyography in an Eriksen flanker task, Rodríguez-Fornells et al. (2002) showed the onset of corrective movements to precede the ERN and start around 68 ms before response. The ERN is a robust marker of error monitoring and it is systematically observed after an error in a variety of tasks conducted under laboratory conditions (Hoffmann and Falkenstein, 2012; Iannaccone et al., 2015; Ora et al., 2015), under more ecological conditions (Padrão et al., 2016), and in situations where the stimulus, and therefore the error, is more or less complex to decipher (Scheffers and Coles, 2000).

### 2.2. Performance monitoring system: other ERP components

#### 2.2.1. Correct-Related Negativity (CRN)

Interestingly, an ERN-like negative ERP peak has also been found in correct trials: the *correct-related negativity* (CRN). The CRN peaks at the same latency—around 100 ms after EMG response or 80 ms after button pressing—and has approximately the same fronto-central distribution as the ERN, but its amplitude is smaller than that of the ERN (Vidal et al., 2000). The CRN is often ignored because performance monitoring-related ERPs are usually measured by differentiating ERP waves of correct and erroneous trials. It was first observed by Hohnsbein et al. (1991), in a choice reaction task (called the N-CR, for Negativity-Choice Reactions), and then replicated on several occasions in other tasks (Falkenstein et al., 2000; Scheffers and Coles, 2000; Luu and Tucker, 2001; Allain et al., 2004; Gentsch et al., 2009; Hoffmann and Falkenstein, 2012).

**Figure 1:**
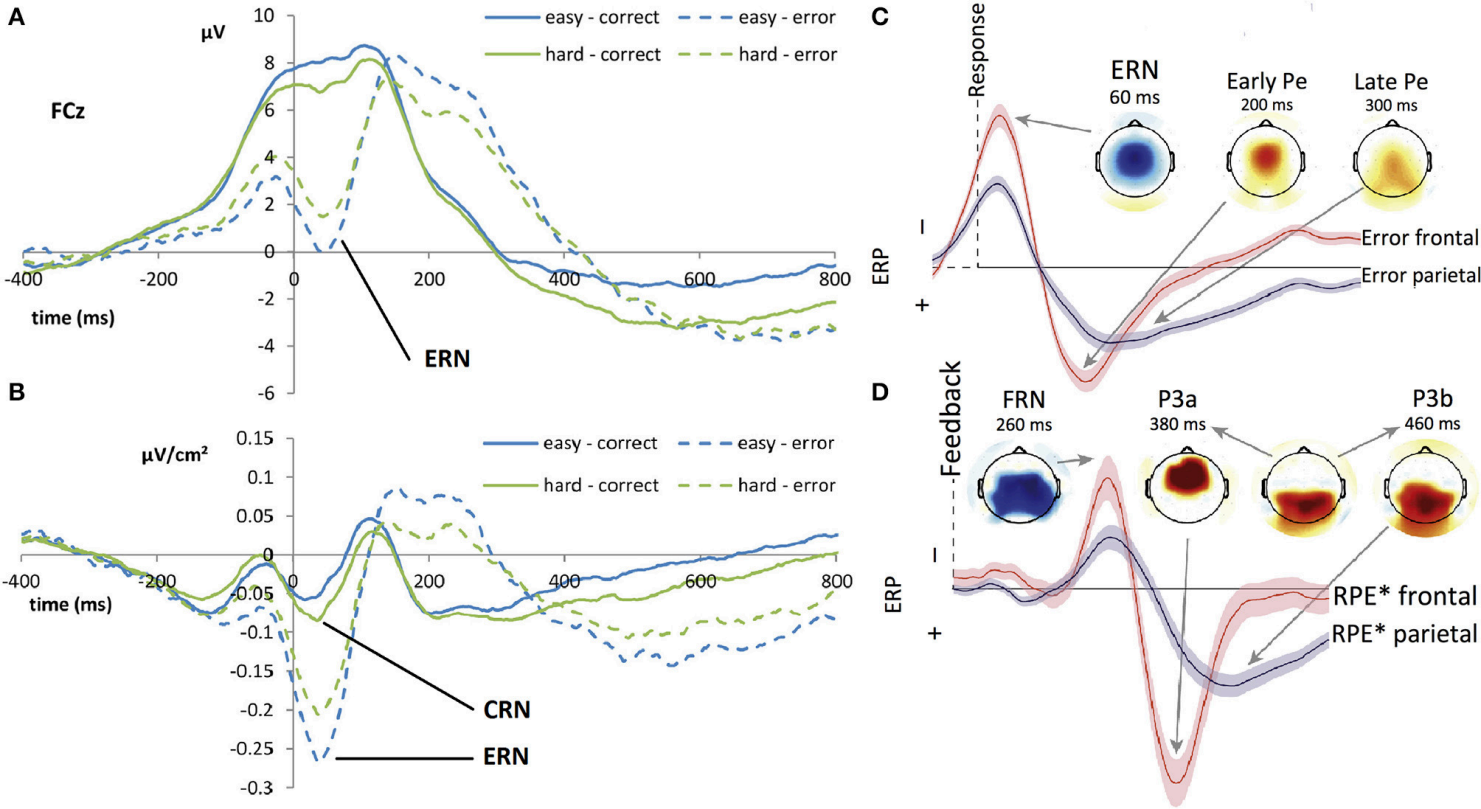
Time course of the event-related potentials (ERP) related to error execution–Adapted from Van der Borght et al. (2016) and Ullsperger et al. (2014b) with their permission. Left panel: **(A)** Grand average ERP waveforms of the CRN (plain line) and ERN (dashed line) at electrode FCz after response execution (time point 0 *ms*) during a forced choice task depending on two levels of difficulty. The ERN is a clearly identifiable negative component, peaking around 80 *ms* after error commission. The CRN associated with correct response is masked by a positive wave. **(B)** Laplacian transformed grand average ERP waveforms of the CRN (plain line) and ERN (dashed line) at electrode FCz after response execution (time point 0 *ms*) during forced choice task depending on two levels of difficulty. The Laplacian transformation removes the positive wave and allows both negative peaks around 80 *ms* after correct and erroneous response to be shown. Right panel: Schematic time-courses of regression weights of models, based on a probabilistic learning paradigm and a flanker-task performed by the same subjects, and their topographies for performance monitoring for erroneous **(C)** response generation, and **(D)** feedback evaluation as revealed by single-trial EEG multiple regression analysis. Both waveforms show a rapid negative potential followed by a frontal positivity and a later more posterior positivity. ERN+Pe time course for erroneous response can be likened to the FRN+P3 time course for negative feedback. RPE^*^ indicates the Reward Prediction Error, multiplied by −1 for better comparability showing correlations with unfavorable outcomes as negative.

According to several studies, the CRN represents a comparison process that happens both for correct and incorrect trials and the greater amplitude of the ERN (in comparison with the CRN) may correspond to the sum of an error-related activity and of the CRN (Falkenstein et al., 2000). Figure 1 shows the time course of the ERN (after errors) and CRN (after correct responses), in monopolar recording and after a Laplacian transformation. Falkenstein (2004) assumes a common superficial cerebral source for both components—ERN and CRN—to which a deeper source is added in erroneous trials. This hypothesis is supported by two studies carried out by Vidal et al. (2003, 2000), who showed a similarity in topographic distributions of the ERN and CRN components using a source derivation method based on the surface Laplacian of the electric potential. Thus, the CRN seems to be part of the performance monitoring system, and is elicited irrespective of the subject performance.

#### 2.2.2. Error positivity (Pe)

In addition to the ERN, a positive-going deflection named *error positivity* (Pe) was identified in centro-parietal locations (maximum at the CPz electrode in the 10–20 system) around 350 ms after an erroneous response (Falkenstein et al., 1991). This potential is linked to the ERN, although it only appears after overt errors (Vidal et al., 2000). In this context, some authors argue that the Pe corresponds to a P3b component specific to errors (Ridderinkhof et al., 2009). Interestingly, the Pe is influenced by both the person's affect (negative or positive) and the saliency of the error-inducing stimulus (Leuthold and Sommer, 1999) but not by the amplitude of the error (Overbeek et al., 2005). These studies also show that genuine errors elicit greater amplitude error positivity than intentional ones. Furthermore, it appears to be consistently modulated by awareness and motivational salience of errors. Regarding these feature differences between the Pe and the ERN, several works suggest that Pe corresponds to a different aspect of error monitoring relying on conscious and volitional processes (Herrmann et al., 2004; Padrão, 2014). Figure 1C shows the time-course and topography of the error positivity.

#### 2.2.3. Feedback-Related Negativity (FRN)

Finally, associated with feedback stimuli following the participants' responses, a negative deflection known as *feedback related negativity* (FRN) is observed. The FRN appears around 250 ms after a “worse-than-expected” feedback onset in the fronto-central regions (electrode FCz with topography similar to that of the ERN and CRN) and interrupts a positive-going wave (most likely the P300; Donkers et al., 2005; Miltner et al., 2004). Figure 1D shows the time-course and topography of the FRN.

Several studies have noted the similarity between the FRN and the ERN and described the FRN as an ERN-like process (Miltner et al., 1997). Until 2003 (Luu et al., 2003), this ERP was called a feedback ERN (fERN) and several groups still use this term (Holroyd et al., 2006; Kam et al., 2012)^1^. First, both the ERN—with its counterpart the CRN—and the FRN are observed, respectively, for all response and feedback types - positive (correct/gain), negative (error/loss) and neutral (uninformative/no loss-no gain) (Holroyd et al., 2006). Second, both components correspond to theta-frequency waves in the spectral domains of EEG activity (Luu and Tucker, 2001; Cavanagh and Frank, 2014). In addition, several authors show that the ERN and FRN originate from the same brain region, as isolated by independent component analysis (ICA): using back projection after an independent component analysis, Gentsch et al. (2009) find both potentials to be elicited by the same set of independent components. This result is supported by other EEG studies (Luu et al., 2003; Nieuwenhuis et al., 2004). Other source localization analyses not based on ICA also identify one or two different generators situated roughly in the same locations for both components (Dehaene et al., 1994; Miltner et al., 1997). Although the latency differs between the ERN (80 ms post-response) and the FRN (250 ms post-feedback), it could be due to the time necessary to process the feedback stimulus in the case of the FRN. The literature often suggests that the ERN and FRN are elicited by the same evaluative system: one when an action is produced, and the other one when one observes the consequence of such action. Nevertheless, this conclusion is still debated. One counter-argument was presented by Donkers et al. (2005), who showed that the ERN and FRN recorded respectively in a modified flanker task and a time estimation task, were not statistically correlated.

Interestingly, several authors claim that both the ERN and the FRN depend neither on the stimulus nor on the modality of the effector (Holroyd and Coles, 2002; Ullsperger et al., 2014b). However, this last point is controversial: other authors claim that the amplitude of the FRN is modulated by the output modalities—somatosensory, visual, auditory (Miltner et al., 1997)—and the attributes of the (feedback) stimulus, as opposed to the amplitude of the ERN (Holroyd and Coles, 2002; Elton et al., 2004; Liu and Gehring, 2009; Gehring et al., 2011). This last effect could be explained by a difference in the processing duration of different modalities or attributes of the feedback stimulus. Table 1 summarizes the characteristics of all of the ERPs associated with performance monitoring, and Figure 1 reflects their time course and topographies.

**Table 1:** Comparison of the characteristics of performance monitoring ERPs.

**Characteristics**	**CRN**	**ERN**	**FRN**	**Pe**
Latency	50–100 ms post-response	50–100 ms post-response	250–300 ms post-feedback	350–500 ms post-response
Valence	Negative	Negative	Negative	Positive
Maximum amplitude (absolute value)	5 μV	15 μV	15 μV	10 μV
Peak activity in the 10–20 system	FCz	FCz	FCz	CPz
Underlying frequency	θ wave	θ wave	θ wave	δ wave
	Hohnsbein et al., 1991; Vidal et al., 2000; Luu and Tucker, 2001	Falkenstein et al., 1991; Gehring et al., 1993; Luu and Tucker, 2001	Miltner et al., 1997; Donkers et al., 2005; Cavanagh and Frank, 2014	Falkenstein et al., 1991; Overbeek et al., 2005

The various components of the performance monitoring system have been studied extensively, and this led to the characterization of a wide range of ERP features. Nevertheless, the relationship between the various ERP components remains unclear. Studying more specifically the brain activity underlying these ERP may help to further characterize such processes.

### 2.3. Performance monitoring system: neural bases

The discovery of the ERN was rapidly followed by studies focusing on the localization in the brain of the performance monitoring system using various source analysis methods (Grandori et al., 1990)^2^. Several studies performed with EEG (intracranial EEG: Bonini et al., 2014; surface EEG: van Schie et al., 2004; Roger et al., 2010), with fMRI (Ullsperger et al., 2007; Desmet et al., 2014; Cracco et al., 2016) or coupled EEG-fMRI (Debener, 2005; Iannaccone et al., 2015) suggest that the brain source of the ERN component is located within the pMFC (posterior Medial Frontal Cortex). More specifically, the Rostral Cingulate Zone (RCZ), which spreads over the Anterior Cingulate Cortex (ACC–involved in autonomic and higher-level functions) and the pre-Supplementary Motor Area (preSMA–involved in volitional stimulus-cued movements), would be the most likely source for this component during self-monitoring of error response^3^. In addition to the ACC, the anterior insula and/or frontal operculum have also been shown to play a role in performance monitoring and might be involved in the generation of the ERN (Ullsperger et al., 2010; Amiez et al., 2016; Bastin et al., 2017).

The orbito-frontal cortex (OFC) has also been proposed as a putative generator of the ERN (Brázdil et al., 2002; Turken and Swick, 2008). Using intracranial recordings, these studies show that neuronal assemblies in the OFC generated the same type of error potentials as the scalp ERN. Moreover, in the study by Turken and Swick (2008), the ERN amplitude appears to be reduced when the OFC is damaged after a lesion. Nevertheless, these results have also been questioned by Ullsperger et al. (2002), who observed no difference in the ERN amplitude or topography in an OFC-lesioned group, when compared to an age-matched control group.

Finally, although the brain source of the Pe ERP component (maximum in centro-parietal regions at CPz) is still debated, most studies converge toward a generation of the error positivity in the rostral part of the ACC (rACC; Falkenstein et al., 2000; Van Veen and Carter, 2002), which is an area that is often involved in affective processing. Although close in location, this brain source would be different from that generating the ERN and FRN.

The fact that the functional characteristics of the ERP components associated with performance monitoring are different, whereas their anatomical substrates seem to be similar, raises the question of the functional role of each of these in performance monitoring.

### 2.4. Performance monitoring system: functional theories

The role of the ACC in high-level cognitive processing and the functional characteristics of the ERN have led several authors to define various functional theories to explain this ERP. A short description of those theories is presented hereafter (for more details, see Rodríguez-Fornells et al., 2002; Gehring et al., 2011; Ullsperger et al., 2014a).

The first theoretical model proposed is the *Mismatch hypothesis* or *Comparator model*. It asserts that the ERN is not correlated to sensory or proprioceptive information resulting from the initial erroneous movement. Instead, the ERN would involve an efferent copy of the motor command (central monitoring as opposed to information from the moving limb) and would be elicited whenever there is a mismatch between the intended, correct response and the actual, incorrect response (Cooke and Diggles, 1984; Falkenstein et al., 1991; Gehring et al., 1993). The issue with this theory is that we also know that there are ERN-like components for correct trials and after feedback: respectively, the CRN and FRN described earlier. Thus, an ERN-like component is also present when there is no mismatch between the correct and the actual response of the participant.

To take into account these results, Holroyd and Coles (2002) proposed an extension of the mismatch hypothesis: the *Reinforcement-learning* theory (RL). This theory states that motor controllers (such as nociceptive or limbic sources) process the stimulus and send information to the ACC, which acts as a control filter for planning and executing motor behavior. While this process is taking place, the activity of ACC and motor controllers would be modulated by mesencephalic dopaminergic neurons from basal ganglia. The signal would be either positive or negative (respectively, if an event is better or worse than expected) and would correspond to increases or decreases in the phasic activity of the dopaminergic neurons. A negative signal would correspond to an ERN. The mesencephalic dopaminergic neurons would also auto-regulate themselves by sending negative feedback to the basal ganglia^4^.

While the RL theory can now explain the presence of the FRN, it does not account for the signal observed for correct responses (the CRN component). Thus, another extension to the theory was recently proposed: the *Prediction of Response Outcome* theory (PRO; Alexander and Brown, 2011). This theory does not assume a comparison of expected responses with efferent motor output, but rather relies on an actor-critic framework of prediction based on past experience. The major difference with the previous theories is the absence of valence (positive or negative signal). This theory can thus account for a negative deflection for correct responses (the CRN component). Both RL and PRO theories explain the functional significance of the ERN and FRN components, which would represent error signals that could trigger learning to avoid repeating the same errors (Holroyd and Coles, 2002). Further study tends to support this assumption and shows that the FRN is elicited when a feedback stimulus deviates from expectations or predictions and when that deviation corresponds to the worst case scenario: a positive feedback can lead to an FRN (Donkers et al., 2005; Holroyd et al., 2006).

Other theories have also been proposed. After the RL theory, one of the most widely accepted theories is the *conflict-monitoring* theory. This theory asserts that the ERN is elicited whenever there is information processing conflict. The ACC would cancel out the expected brain response and the ERN would correspond to cases in which the incorrect response is not successfully overridden by the ACC (Botvinick et al., 1999, 2001). This theory explains well the CRN and most of the error-related experimental results. However, experimental studies show no modulation of the ERN with the level of conflict (Scheffers and Coles, 2000). Moreover, this theory was shown as unfit by Burle et al. (2008) in a study in which they compared experimental results with simulated ones obtained using an artificial neural network based on the conflict-loop theory^5^.

Although no consensus has been reached yet, several theories also attempt to explain the functional role of the error positivity (Pe). The first theory assumes that the Pe component reflects a delayed parietal P300 wave related to the amount of information extracted from the error, or would correspond to a late P3b specific to the error (Leuthold and Sommer, 1999). This theory is supported by several experimental results, such as the fact that the amplitude of the Pe, as for the P300, is the same between childhood and early adulthood but is reduced during late adulthood (Overbeek et al., 2005). However, Falkenstein et al. (2000) discarded this hypothesis by showing that, in some cases, the P300 amplitude is modified without any impact on the Pe.

It was also proposed that the Pe component would reflect the emotional or subjective error assessment (Van Veen and Carter, 2002). Indeed, its amplitude is modulated by emotions: for example, a negative affect decreases the amplitude of the Pe (Hajcak et al., 2004). Moreover, some studies found that the amplitude of the Pe varies with the number of participants' errors. This theory would be supported by the fact that participants become less emotionally affected with an increasing number of errors (Falkenstein et al., 2000; Mathewson et al., 2005). A study by Stemmer et al. (2001, Figure 3) also shows that Pe is only observed for genuine errors and not for intentional errors. However, most of those results remain disputed, and some studies showed contradictory results. The main consensus states that the Pe component reflects error awareness (Nieuwenhuis et al., 2001; Rigoni et al., 2015). Although this characteristic was initially attributed to the ERN, Scheffers et al. (1996) and later Vidal et al. (2000) showed that the Pe was a more adequate candidate using EMG measures to record both *overt errors* (the EMG is above the motor command threshold and the subject presses the corresponding button), and *covert errors* (there is an above-threshold EMG for the erroneous response, but the subject actually chooses not to press the button and the error is not acted upon). While the Pe component is only observed for overt errors, the ERN is present for both types of errors.

### 2.5. Conclusive remarks about performance monitoring

In conclusion, the mechanisms underlying monitoring of self-performance are well-documented. The large collection of studies in various research fields provides us with an in-depth understanding of this process. Several markers of performance monitoring have been identified: (i) *the error related negativity* (ERN), the principal component elicited for error execution; (ii) *the correct-related negativity* (CRN), elicited for the performance monitoring of correct actions; (iii) *the feedback-related negativity* (FRN), elicited by any type of feedback and (iv) *the error positivity* (Pe), an error-specific component that is only present for overt errors.

More recently, several authors have tried to extend those results and the associated theories to the monitoring of others' performance, i.e., another human or artificial agent. Indeed, by observing others, we are able to learn from their mistakes (in the same way as babies learn by observing their parents) and the Reinforcement-Learning theory (RL) can easily be extended to include other agents. The following section reviews our knowledge about error monitoring of human agents and automated systems.

## 3. Performance monitoring and error observation

**Figure 2:**
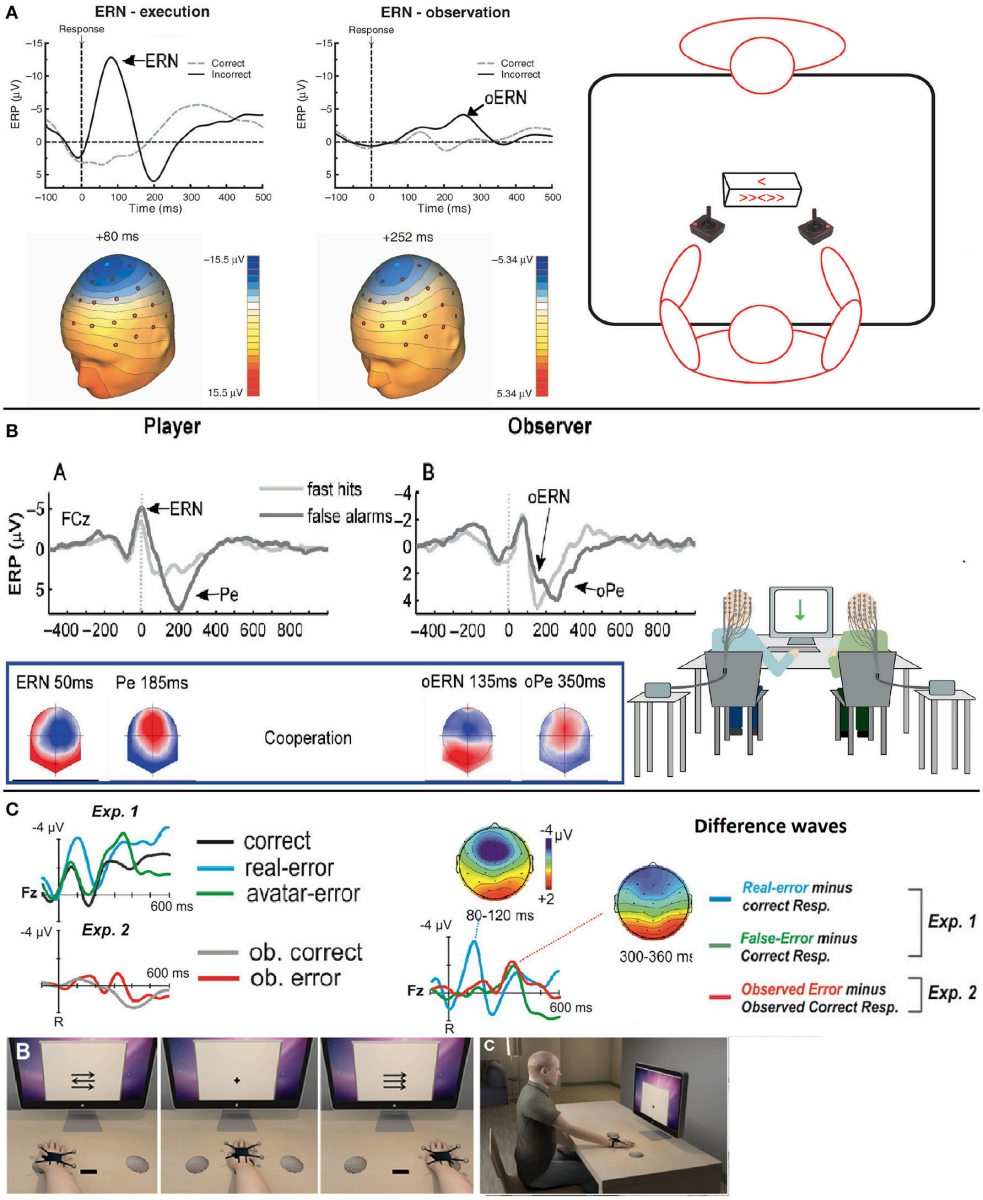
Task schematic, time course and topographies of the error-related potentials for correct responses and errors during other's or system supervision–Adapted from van Schie et al. (2004), Koban et al. (2010) and Padrão et al. (2016) with their permission. **(A)** Grand average ERP waveforms obtained during execution (ERN) of an error and observation (oERN) of anothers error at Cz electrode when the observer is seated in front of the performer. There is a negative wave peaking in both cases after an error is produced. Topographies are also similar. **(B)** Grand average ERP waveforms of the ERN/Pe and oERN/oPe at the FCz electrode for execution and observation of errors and correct responses when the observer is seated next to the performer. Topographies are similar between the ERN and the oERN, and between the Pe and the oPe. **(C)** Top left–Grand average ERP waveforms related to correct responses, performer errors and avatar error during a 1PP virtual reality monitoring task (Exp. 1, as represented by Illustration B), and grand average ERP waveforms related to observation of correct responses and errors of an avatar during a 3PP virtual reality monitoring task (Exp. 2, as represented by Illustration C). Top right–We observe an ERN at the Fz electrode after error commission compared to a correct response (blue difference wave) and an oERN after both avatar errors (i.e., system malfunctions) and 3PP observed errors (green and red difference waves respectively). Bottom–Illustration of a 1PP (B) and a 3PP (C) virtual reality monitoring tasks.

Two types of non-self agent supervision can be distinguished: the supervision of a human agent, which may involve social and emotional components, and the supervision of an artificial agent. The state of the art for both types of interaction is described below, and Figure 2 shows the event-related potentials' time course and topographies associated with error observations.

### 3.1. Human agent error

Detecting others' errors is crucial for functions such as anticipation of others' actions, or teaching. The first neurophysiological correlates of this cognitive process were identified by Miltner et al. (2004), using experimental protocols similar to those used for error execution monitoring (4-way choice reaction tasks). They recorded a negative wave peaking at around 230 ms after the observation of an error performed by another participant, at central locations (electrode Cz). Interestingly, this negative wave interrupts a positive-going wave peaking at around 300 ms after observation of the error and could therefore be likened to the feedback-related negativity peak described previously.

This negative wave is called the “observation ERN” (oERN as opposed to “response ERN”)^6^, it peaks at around 230–250 ms, and has been reported in several studies with different tasks, such as Eriksen flanker tasks (van Schie et al., 2004; Carp et al., 2009; de Bruijn and von Rhein, 2012), gambling tasks (Yu and Zhou, 2006; Marco-Pallarés et al., 2010) and, with earlier latencies, Go/NoGo tasks (135–150 ms; Bates et al., 2005; Koban et al., 2010). Figure 2 shows the time course of this potential in various task configurations. Some studies also report a positivity following this negative wave (Carp et al., 2009; Koban et al., 2010). Like in the case of the oERN with the ERN component, this positive wave is similar to the Pe component. This component is called the “observation Pe” (oPe as opposed to the “response Pe”), it arises between 250 and 500 ms after the observation of the agent's error and has similar topographies to those of the Pe, as suggested by Koban et al. (2010) (see Figure 2B for a comparison of the Pe and oPe).

Finally, an oERN-like ERP has been found for correct trials: this small negativity peaks at approximately the same latency as the oERN (around 230 ms, −1.3μV) and with the same topography (at fronto-central sites Fz, FCz, and Cz) as the oERN/FRN (Carp et al., 2009). This negative component could be related to the CRN component observed during the execution of correct trials during self-monitoring.

The similarity between the components associated with the monitoring of our own performance and the monitoring of others' performance is strengthened by an equivalent amplitude modulation of the ERN and oERN with the valence of responses (Yu and Zhou, 2006; Marco-Pallarés et al., 2010). In addition, no amplitude modulation of the FRN (self-monitoring) and the oERN/FRN (others' supervision) is observed with the value of the gain or loss in gambling tasks (Yu and Zhou, 2006). Finally, source localization in EEG and fMRI studies both locate the generator of the oERN/FRN in the same brain area as that identified for the ERN and FRN components (van Schie et al., 2004; Desmet et al., 2014). In conclusion, the same performance monitoring system seems to be involved during the monitoring of self-executed errors and the monitoring of others' errors.

Interestingly, performance monitoring of others' action is also modulated by various psychosocial parameters. One parameter is the intention which may be attributed to the person performing the action. Desmet and Brass (2015) used an observational task with three levels of intentions and show that the observation of unusual intentional actions activates the anterior medial prefrontal cortex (aMPFC) more, while the posterior medial prefrontal cortex (pMPFC) is more active during the observation of unusual accidental actions. Another modulating parameter is interpersonal similarity, based on participants' beliefs and opinions, which correlates negatively with the oERN and positively with the oPe (Carp et al., 2009). Moreover, other parameters like the sense of agency, social context and empathy toward the performer have an effect on the activation of the posterior medial frontal cortex (pMFC)—including the RCZ and pre-SMA—which is involved in the monitoring of others errors. They also modulate the activation of both the anterior insula and the cerebral network associated with error (Cracco et al., 2016)^7^. Considering these various results, it is possible that the performance monitoring system differs between supervision of a human performer and supervision of an automated system.

### 3.2. System error

Studies about system error/performance monitoring found in the literature can be divided into two groups: (i) studies of system malfunctions triggered by human agent actions (Gentsch et al., 2009; Ullsperger et al., 2010; Padrão et al., 2016); and (ii) studies about the observation of system errors without any action required from the human agent (Desmet et al., 2014; Pavone et al., 2016). The system used may be a computer, a first-person perspective (1PP^8^) or third-person perspective (3PP) virtual reality avatar (i.e., “[…] computer generated visual representations of people or bots”; Nowak and Rauh, 2005) or a Brain-Computer Interface (BCI, i.e., systems “[…] utilizing the brain signals in a man-computer dialogue”; Vidal, 1973). In all cases, several performance monitoring tasks, from the most classical ones (e.g., the Eriksen flanker attention task, moving cursor task) to more ecological ones (e.g., video clip observation, object grasping) were used in fMRI studies (Ullsperger et al., 2007; Desmet et al., 2014) or EEG studies (Ferrez and Millán, 2005; Ferrez and Millán, 2008; Gentsch et al., 2009; Kreilinger et al., 2012; Pavone et al., 2016).

Irrespective of the method used to study the monitoring of an automated system's performance, at least one of the two event-related potentials classically obtained during the supervision of others' errors was observed in most studies, with similar latencies and topographies: an oERN/FRN and an oPe peaking respectively between 250 and 270 ms and between 350 and 450 ms post-error in fronto-central regions (Ferrez and Millán, 2005; Ferrez and Millán, 2008, see Figure 2C for the time course of these ERPs). These electrical potentials are found to be extremely robust and stable over time—when measured more than 600 days apart—in Chavarriaga and Millán's (2010) BCI study. In addition, based on frequency band analyses, Pavone et al. (2016) showed that the amplitude of oERN/FRN was positively correlated with an increase in theta frequency bands, the same frequency band that is involved in ERN and FRN generation. Interestingly, in most studies (Ferrez and Millán, 2005; Chavarriaga and Millán, 2010; Padrão et al., 2016; Pavone et al., 2016), the authors found a new monitoring-related ERP: a negative wave peaking at fronto-central locations between 400 and 550 ms after the observation of a system error. This potential, initially called *interaction Error-related Potential* (ErrP; Ferrez and Millán, 2005), is believed to be related to the N400 ERP. The N400 is usually observed when semantic aberrations occur and it peaks around 450 ms after stimulus onset at centro-parietal locations (Kutas and Hillyard, 1980). However, Balconi and Vitaloni (2014) showed that this ERP is also observed after an incongruous ending in a sequence of movements at more fronto-central and temporo-parietal locations. The presence of the N400 in system error monitoring is therefore not surprising.

Source localization of the ERPs generated during system supervision points to pre-SMA and RCZ brain generators (Ferrez and Millán, 2008). This result is supported by an fMRI study by Ullsperger et al. (2007) who showed that the RCZ is similarly activated during system error monitoring and monitoring of our own errors. When comparing the two types of task, only sensorimotor areas appear to be predominantly more activated when we perform our own errors. In addition, a study by Desmet et al. (2014) showed that the same brain regions—particularly pre-SMA, but not RCZ—are activated during the supervision of human and machine errors.

The theory that system performance monitoring is similar to that of human agents, with no need for motor action, was initially proposed by Holroyd et al.'s (2004) and tends to be supported by an EEG study carried out by Yeung et al. (2004). The authors used a gambling task in which an observer passively monitored computer-generated outcomes (gain or loss). They detected an FRN component for the observer after feedback, but with no movement. Likewise, a follow-up study by Donkers et al. (2005) showed a mediofrontal negativity (MFN) positively and significantly correlated to the FRN in case of a slot-machine task, with outcomes that are not contingent upon recent actions.

Table 2 summarizes the results obtained in terms of electrical potentials and localization for the supervision of another agent's errors, human or artificial. Altogether, the results that we have just reviewed seem to support Holroyd et al. (2004) hypothesis. Nevertheless, further directions need to be addressed.

**Table 2:** Comparison of ERP components and their generators for agent error supervision.

		**Human agent error**	**System error**			
			**Error observation**	**System malfunction**		
				**Avatar**	**BCI**	**Computer**
ERPs	oERN/FRN	✓ van Schie et al., 2004	✓ Pavone et al., 2016	✓ Pavone et al., 2016	✓ Ferrez and Millán, 2008	✓ Gentsch et al., 2009
	oPe	✓ Carp et al., 2009	✓ Padrão et al., 2016	✓ Padrão et al., 2016	✓ Ferrez and Millán, 2008	?
	N400	?	✓ Pavone et al., 2016	✓ Padrão et al., 2016	✓ Ferrez and Millán, 2008	✓ Padrão et al., 2016
fMRI/Source localization	preSMA	✓ Desmet et al., 2014	✓ Desmet et al., 2014	?	✓ Ferrez and Millán, 2008	X Ullsperger et al., 2007
	RCZ	X Desmet et al., 2014	X Desmet et al., 2014	?	✓ Ferrez and Millán, 2008	✓ Ullsperger et al., 2007

Legend–✓: tested and found; X: tested and not found; ?: not tested

## 4. Further directions

An interesting application of the role of performance monitoring in system supervision stands in the out-of-the(-control)-loop (OOL) performance problem (Endsley and Kiris, 1995). The OOL performance problem represents a key challenge for system designers as it is likely the cause of several accidents (Endsley, 1996; see other examples in Sparaco, 1995; Board, 1997; Lee and Sanquist, 2000). One important behavioral aspect of the OOL performance problem is the insufficient monitoring and checking of automated functions by human operators (Kaber and Endsley, 1997). Interestingly, a recent study by Kam et al. (2012) showed a smaller FRN when subjects were mind-wandering, thus not in the control loop in a time-estimation task. This study shows for the first time a relationship between the OOL phenomenon and the performance monitoring functions. Regardless of the origins of the monitoring performance dysfunction, there is a need to characterize the OOL performance problem to better understand and counteract this phenomenon. Studying the origins of individuals' errors, based on data and methodologies in cognitive neurosciences would allow some aspects of system performance monitoring in different task contexts to be generalized (Sarter and Sarter, 2003; Fedota and Parasuraman, 2010; Johnson and Gulbinaite, 2013). Although this is still being debated, some studies suggest that the observation of our own errors (error execution monitoring) and those of others (error observation monitoring) would engage the performance monitoring system in similar ways (Miltner et al., 2004; Carp et al., 2009; Jääskeläinen et al., 2016, to cite a few). However, several limits remain to be able to generalize such results to the supervision of system errors. The following section will review the relevant literature in order to better understand how one supervises a system, and whether or not all of this knowledge may enable us to tackle monitoring problems.

### 4.1. Artificial agent and performance monitoring

Although neural correlates of performance monitoring seem to be similar during system error supervision, supervision of other human agent's errors and error execution monitoring, important research remains to be done.

A first concern is the role of the motor component in the performance monitoring function. Research on error observation has mainly focused on human agents or human-like agents (avatars). In these studies, the movements performed by the performer or avatar are biological movements. Likewise, the BCI community went further into the study of performance monitoring when they looked at the supervision of automated systems. However, in every BCI study, the subject was still either required to perform a movement or asked to imagine it (Kreilinger et al., 2012). Therefore, the motor component and/or its biological properties were still present. However, one of the major differences between the current observation studies and system supervision is the absence of movements. When observing another human agent or avatar, observers may relate to the agent's movement. Also, the two main theories for the ERN component—i.e., reinforcement learning and conflict monitoring—both rely on motor activation: reinforcement learning states that the input of the performance monitoring system is a copy of motor plans, and conflict monitoring is based on overriding motor response. Then, we also know that BCI robots use simple anticipatory movements that can be interpreted by an observer according to kinematics laws. Also, it was shown that one was able to anticipate whether another experimenter was in cooperation or competition with him/her based not only on real movement observation (video watching of a performer) but also on simple point light movement observation based on kinematics laws (Manera et al., 2011). Therefore, all systems previously studied show anticipatory movements. However, in the case of system supervision, anticipation of movements is not applicable given that there are no movements performed by the system. Finally, the source of the performance monitoring system is assumed to be located in the RCZ, which spreads over the pre-SMA and ACC (Ridderinkhof et al., 2004, see Figure 2), and the role of the pre-SMA in various motor functions is well documented.

A second critical concern relies on the psychosocial aspects of the human-machine interaction. We have argued in the previous sections that performance monitoring depends on the purpose as well as on the emotions, affect, or social context that the operator is immersed in. Several results point out discrepancies between observing the errors of another human agent and observing the errors of a machine/automated system. For example, we know that empathy acts on performance monitoring. Yet it was found that the less a system, or a robot, is humanoid, the less we feel empathy for it (Riek et al., 2009). Moreover, comparing trust in humans and automated systems, Lewandowsky et al. (2000) found that humans operators tend to trust task allocation performed by an automated system more than task allocation performed by a human collaborator. Such overconfidence can affect the monitoring of the system's outcome in the case of human-machine interactions. More interestingly, several studies show that authorship, as measured by agency, is attenuated in the case of human-machine interaction, both because it is more difficult to attribute intentionality to an artificial agent than it is to another human agent (Wohlschläger et al., 2003; Crivelli and Balconi, 2010) and because human-machine interaction creates a diffusion of responsibility (Beyer et al., 2017). Such a decrease in the sense of agency is reflected in the ERPs' amplitude: there is a lower FRN and P300 in the case of a bad outcome (Li et al., 2010; Beyer et al., 2017). Even though the performance monitoring system seems to be modified by the introduction of another agent, the loop involving control, responsibility, and monitoring is not clearly defined at the moment. These different issues make the comparison between performance monitoring of human agents and of automated systems uncertain.

### 4.2. Performance monitoring and system supervision: measurement problem and ecological validity

Measurement issues concerning performance monitoring must be pointed out. Several performance monitoring studies are ERP studies. The potentials recorded depend on several parameters, defined by researchers based on their experience or on conventions. One difficulty for the ERN and CRN is entailed by the fact that they are response-locked fronto-centrally distributed potentials. Thus, motor activity may interfere with their analysis and, more precisely, with the definition of a baseline before the response. Most studies have used conventional ERP baselines: 50, 100, or 500 ms prior response (Gehring et al., 1993; Scheffers and Coles, 2000; van Schie et al., 2004; Koban et al., 2010; de Bruijn and von Rhein, 2012; Padrão et al., 2016), and from 300 to 200 ms or 500 to 100 ms prior response (Bates et al., 2005; Burle et al., 2008). These baselines are widely used for stimulus-locked ERP analysis, but present the inconvenience of falling right into the time period of motor activity for response-locked ERP studies. This issue is particularly difficult to overcome in ecological studies, like those found in the human-machine interaction field. Indeed, the trigger for the response can be more or less precise in these studies, and it can correspond to a time period rather than a time point. Thus, other less conventional baselines should be used. For example, Miltner et al. (2004) chose to set average ERPs to 0 μV at time point –50 ms for response ERPs and at time point –200 ms for feedback ERPs. On another level, a few authors have used frequency or time-frequency analyses (Luu and Tucker, 2001; Cavanagh and Frank, 2014; Padrão et al., 2015). These types of studies give more information about the processes underlying performance monitoring. Knowing the frequency of each ERP wave can tell us whether or not they are related. It can also give a better time-scale of the performance monitoring system, or help us to give more complete functional theories about this system (see the RL theory description). They are also interesting because they can be used to study more complex errors with, for example, several degrees of error (e.g., in aircraft or car simulations). The study of performance-monitoring-related potentials time-locked to a response (CRN, ERN, and Pe) or a stimulus (FRN, oERN, and oPe) is quite restrictive, since it requires a special trigger in time to identify these potentials.

Another issue arises from the use of a grand average to define the difference wave in ERP analysis. People usually interact with and supervise highly reliable systems. With such systems, only a few errors are expected, and classical grand average is not suitable. A solution to this limitation is reflected in the use of trial-by-trial analysis. Such an analysis was performed by Pardo-Vazquez et al. (2014) on performance monitoring ERPs. Another limitation comes from the difference wave measure: it is reductive and often masks processes going on, especially since the ERN overlaps at least one, or more components (Gehring et al., 2011, see Figure 10.21). We have mentioned previously that the use of difference waves masked the CRN in correct trials. Falkenstein et al. (2000), Luu et al. (2000) and Vidal et al. (2000) were the first to show the existence of a negative peak in correct trials. They compared erroneous and correct trials using either an averaged reference or estimation of the surface Laplacian. The CRN is masked by the conventional mastoid referencing and the difference wave (see Figures 1A,B) for a comparison between grand averages before and after Laplacian transformation). These results led to functional re-evaluation of the definition of the ERN (Vidal et al., 2003). The use of the estimation of the Laplacian enabled a better spatio-temporal analysis of the data. Moreover, using the CRN to assess performance monitoring during reliable system supervision is an interesting line of research. This potential is linked to correct responses, yet corresponds to performance monitoring. The study of its amplitude during automation supervision would enable the difficulty of the small quantity of erroneous trials to be overcome.

Finally, current studies on performance monitoring generally use laboratory tasks. The exact nature of the performance monitoring function in more ecological tasks remains unclear. Although laboratory tasks are goal-directed, the goals are often not comparable to those present in fully automated environments. More ecological studies began to appear in the BCI community for to assess other's or system performance monitoring. Ferrez and Millán (2008) and Desmet et al. (2014) evaluated the role of social context in performance monitoring using recordings of everyday life situations. These studies suggest that the knowledge about performance monitoring can be extended to ecological situations, but additional research is needed in this area.

Using broader analyses allows us to free ourselves from several constraints. Opening our minds to unconventional analyses of brain waves and tasks is also an interesting line of study that deserves further attention. Moreover, it can help to better understand how system supervision can function in applied context, which is an essential next step in automation supervision comprehension.

## 5. Conclusion

After half a century of research on performance monitoring, some pieces of the puzzle are still missing. This vital cognitive function has been studied extensively, enabling for a good definition of its various components in a wide variety of situations. Nevertheless, some aspects of performance monitoring are still understudied, such as error detection in the case of a human-automation interaction, in which the operator supervises the system. Automation of systems and processes has deeply modified our role as actors, and the way in which we supervise systems and interpret their errors and performance. A few open questions remain: is an action required to have good performance monitoring? Does system opacity prevent us from supervising it? Do we need to be socially involved to monitor a system correctly? We need to better understand the neurophysiological correlates underlying supervision, in order to better tackle associated cognitive dysfunctions, like the OOL phenomenon, that appear with increasing automation.

## Author contributions

BS, BB, and AC decided on the review's subject. BS wrote the review and tables. BB, AC, and AD supervised, commented and critically revised the manuscript. All authors substantially contributed to this review and all approved the final version.

### Conflict of interest statement

The authors declare that the research was conducted in the absence of any commercial or financial relationships that could be construed as a potential conflict of interest.

## Footnotes

^1^To avoid any confusion, we will use the term FRN in this paper and not fERN.

^2^For a meta-analysis on the generators of the ERN, see Cieslik et al. (2015).

^3^For a proper description and illustration of these different areas, see Ridderinkhof et al. (2004).

^4^For more information concerning this theory, see Holroyd and Coles (2002), and see Figure 1 of their article for a schematic model.

^5^For a description of this theory, see Carter and van Veen (2007).

^6^This ERP is also sometimes considered to be an FRN, but whether it is an oERN or an FRN is undetermined yet. That is why we will use oERN/FRN in this review.

^7^In this study, the observer stops a square from switching colors whenever he/she wants. This color determines the degree of difficulty that the performer will have to face in a flanker task.

^8^In this case the avatar is just the arm whose movement mimics the movement of the subject's arm. In other cases, the avatar may be seen from a third-person perspective (3PP) where the subject sees the avatar facing him/her—see Figure 1 in the study by Pavone et al. (2016).
